# Integrated nursing and medical management improves outcomes in pediatric lobar pneumonia: a randomized controlled study

**DOI:** 10.3389/fped.2025.1612618

**Published:** 2025-07-28

**Authors:** Yuxiao Hu, Qianli Guo, Xuejiao Liu, Wenshan Lv, Linlin Liu

**Affiliations:** ^1^ Respiratory Department II, Children's Hospital of Hebei Province (Hebei Provincial Clinical Research Center for Child Health and Disease; Hebei Provincial Medical Key Discipline), Shijiazhuang, China; ^2^ Lung Function Laboratory, Children's Hospital of Hebei Province (Hebei Provincial Clinical Research Center for Child Health and Disease; Hebei Provincial Medical Key Discipline), Shijiazhuang, China

**Keywords:** lobar pneumonia, pediatrics, integrated nursing and medical management, pulmonary function, inflammatory markers, satisfaction

## Abstract

**Objective:**

To evaluate the effectiveness of integrated nursing and medical management in children with lobar pneumonia, focusing on symptom relief, pulmonary function recovery, inflammation control, length of hospital stay, and caregiver satisfaction.

**Methods:**

Fifty pediatric patients with lobar pneumonia were randomly assigned to receive either routine nursing care or an integrated medical and nursing intervention. Key clinical outcomes—including the duration of symptoms, pulmonary function indices, inflammatory markers, length of hospital stay, treatment efficacy, caregiver satisfaction, and adverse events—were compared between groups.

**Results:**

Compared with the control group, the observation group demonstrated significantly shorter durations of fever, cough, and pulmonary rales, reduced antibiotic usage, and shorter hospital stays (all *P* < 0.001). Pulmonary function indices improved markedly (*P* = 0.001), and inflammatory markers showed more substantial reductions (*P* < 0.001). The overall treatment effectiveness in the observation group was 100%, with a caregiver satisfaction rate of 96.00% and a complication rate of 8.00%, all significantly better than those in the control group (*P* < 0.05).

**Conclusion:**

Integrated nursing and medical management significantly improves clinical outcomes for pediatric lobar pneumonia, accelerating recovery, enhancing pulmonary function, reducing complications, and increasing caregiver satisfaction. These findings support its broader application in clinical practice.

**ClinicalTrials.gov Identifier:**

https://clinicaltrials.gov/study/NCT06945991, NCT06945991 (16th/April/2025).

## 1 Introduction

Lobar pneumonia remains a leading cause of morbidity and hospitalization among children worldwide, with pediatric patients exhibiting distinct clinical characteristics such as persistent high fever, cough, and pulmonary rales, often resulting in significant impairment of lung function and prolonged recovery periods (1). The condition is predominantly caused by bacterial infections, with common pathogens of community-acquired lobar pneumonia including *Streptococcus pneumoniae* (2), *Klebsiella pneumoniae* (3), and *Mycoplasma pneumoniae* (4). Despite advances in antibiotic therapy, the burden of pediatric lobar pneumonia remains substantial (5). Recent epidemiological studies indicate a rising incidence among children, especially in China, where it constitutes up to 30% of community-acquired pneumonia cases (6–8). Notably, children are more susceptible to severe disease and complications due to their unique anatomical and immunological profiles, underscoring the need for tailored management strategies.

Traditional management typically relies on routine nursing and empirical antibiotic therapy, yet challenges persist. Fragmented care, limited multidisciplinary collaboration, and inadequate family engagement are common issues, often resulting in suboptimal outcomes such as delayed symptom resolution and impaired pulmonary recovery (9, 10). Furthermore, current care models seldom incorporate pulmonary rehabilitation or dynamic inflammatory monitoring—elements increasingly recognized as crucial for improving pediatric outcomes (11, 12).

In recent years, integrated medical and nursing care has emerged as an innovative management model, demonstrating notable advantages in the management of chronic diseases and critical illnesses (13, 14). This approach emphasizes multidisciplinary team collaboration, standardized clinical pathways, and continuous engagement of patients and their families. For example, in adult patients with chronic obstructive pulmonary disease (COPD), community-based integrated care—coordinating pulmonologists, nurses, rehabilitation therapists, and nutritionists—has significantly reduced hospital stays and improved pulmonary function (15). However, research on integrated care in the context of pediatric lobar pneumonia remains limited, particularly with respect to high-quality evidence derived from randomized controlled trials (RCTs). Moreover, existing studies primarily focus on short-term clinical outcomes, with insufficient systematic evaluation of long-term effects on pulmonary function recovery, inflammation control, and family-centered interventions.

This single-blind randomized controlled trial assessed whether integrated nursing and medical care, compared to conventional treatment, could enhance pulmonary function, reduce hospital stay, and lower inflammatory markers in children with lobar pneumonia, while also evaluating clinical feasibility and caregiver satisfaction. We hypothesized that multidisciplinary collaboration and family engagement would accelerate symptom resolution, improve respiratory recovery, and decrease complication rates—offering evidence to optimize pediatric pneumonia management and inform efficient healthcare resource allocation.

## 2 Methods

### 2.1 Study design

This study was a single-blind, parallel-group, repeated-measures randomized controlled trial conducted from January 2023 to January 2025 in the Department of Respiratory Medicine at a hospital in Hebei, China. Participants randomly assigned to the intervention group received both integrated medical and nursing care in addition to routine nursing, while those assigned to the waitlist control group received only routine nursing care. Outcome measures were assessed at baseline (T0) and at discharge (T1).

### 2.2 Participants

Pediatric patients diagnosed with lobar pneumonia were recruited from the Department of Respiratory Medicine at a hospital using convenience sampling. Informed consent was obtained from the child's legal guardians.

#### 2.2.1 Inclusion criteria

(1) Met the diagnostic criteria for lobar pneumonia, presenting with symptoms such as fever, cough, and pulmonary rales, and confirmed by laboratory tests and chest x-ray; (2) Aged between 1 and 12 years.

#### 2.2.2 Exclusion criteria

(1) Presence of other concurrent respiratory diseases; (2) Congenital or acquired airway malformations, or airway foreign bodies; (3) Impaired hepatic or renal function; (4) Presence of other systemic diseases; (5) Cognitive impairment.

### 2.3 Sample size

Sample size calculation for the randomized controlled design was performed using G*Power software (version 3.1.9.4). Sample size estimation was based on Li et al. (16), who reported significant improvements in FEV and FVC with integrated care in severe pneumonia. Using FEV as the primary outcome, a Cohen's d of 0.83 was applied to reflect the large observed effect size. Based on a significance level of 0.05, a power of 0.80, and two groups, the calculated sample size was 48 participants. To account for an anticipated 15% attrition rate, 56 participants were enrolled.

### 2.4 Randomization and blinding

Participants were randomly assigned to the intervention or control group using block randomization (block size of eight) generated online (http://www.randomizer.org). Allocation was concealed in advance by an independent assistant using sealed envelopes, which were opened sequentially after baseline data collection. Outcome assessors were blinded to group assignment.

### 2.5 Implementation of the intervention

In the control group, children received standard inpatient care following current pediatric community-acquired pneumonia (CAP) guidelines. This included: Medication management: Weight-based antibiotics (typically amoxicillin or ceftriaxone as per IDSA/PIDS guidelines), along with antipyretics and intravenous fluids guided by physician orders. Therapy was reviewed on Days 1, 3, and 7. Respiratory support: Nebulized inhalation (e.g., β_2_-agonists or saline) administered twice daily for approximately 15 min, with vital signs monitored throughout. Caregivers were taught chest physiotherapy (back patting) upon admission, with nursing staff ensuring daily execution and documentation. Environmental and ward measures: Daily ward air disinfection using ultraviolet lamps (30 min each morning) and regular ventilation per hospital protocol. Monitoring and assessment: Nurses performed twice-daily checks of SpO_2_, respiratory rate, heart rate, temperature, and conducted routine lab tests (CBC, CRP, PCT) and chest imaging as ordered. Any SpO_2_ < 94% or respiratory distress triggered immediate physician notification. Nutrition support: Within 24 h of admission, a dietitian assessed patients and prescribed age-appropriate nutrition. Nurses recorded daily intake/output and monitored hydration status. Nurse-physician communication: Routine ward rounds occurred twice daily, with verbal handovers at each shift. Nurses conveyed abnormal findings promptly to physicians, though no formal multidisciplinary protocol was in place.

The observation group received integrated medical and nursing care in addition to routine care during hospitalization, representing a coordinated intervention that integrates multiple medical resources. This integrated care included two components: (1) A multidisciplinary team collaboration for a two-week inpatient health management period (17, 18); (2) Family intervention (19). Experts, including pulmonologists, clinical nurses, rehabilitation therapists, and nutritionists, reviewed the draft of the integrated medical and nursing intervention plan. Feedback from the experts indicated that the plan was simple, easy to understand, and applicable to children with lobar pneumonia at this hospital. Consequently, the intervention plan was adopted without any changes. The components of the plan are outlined as follows.

1. Multidisciplinary Team Collaboration for Health Management (Supplementary Table S1)
2. Family intervention (Supplementary Table S2)

### 2.6 Data collection

Researchers collected data from the hospital medical record database, including information such as gender (male, female), age (years), disease duration (days), lung lobe infection sites, and clinical symptoms. The clinical symptoms recorded included: Duration of fever (days), Duration of cough (days), Duration of lung crackles (days), Duration of antibiotic use (days), Duration of hospitalization (days).

Lung function tests [FEV_1_ (L), FVC (L), and FEV_1_/FVC (%)] and laboratory inflammatory markers [CRP (mg/L), WBC (×10^9^/L), PCT (ng/ml), and LDH (U/L)] were measured on the day of admission and the day before discharge. Data were collected accordingly.

According to the PIDS/IDSA CAP guidelines for children (20), efficacy was classified as cure (complete symptom resolution and radiological recovery), improvement (partial symptom relief and lesion absorption), or ineffective (no significant change). The total effective rate included both cure and improvement. Family satisfaction was rated on a four-point scale, and adverse events and complications were recorded throughout the intervention.

### 2.7 Statistical analysis

SPSS 26.0 (IBM Corp., Armonk, NY, USA) statistical softwares were used for data analysis. The α level was 0.05 by bilateral statistical test. If the *P*-value is less than 0.05, it is considered significant. The Shapiro–Wilk test and Paired *t*-test were used to evaluate the normality of continuous data. The normal distribution of measurement data was expressed as. Non-normally distributed continuous data were expressed as median (minimum, maximum), and the Mann–Whitney *U*-test was used for comparisons between groups. Independent sample *t*-test was used for comparison between groups. The statistical data were expressed by [case number (%)] and χ^2^ test was used between groups. *P* < 0.05 was considered statistically significant.

## 3 Results

### 3.1 CONSORT flowchart of the study

**Figure 1:**
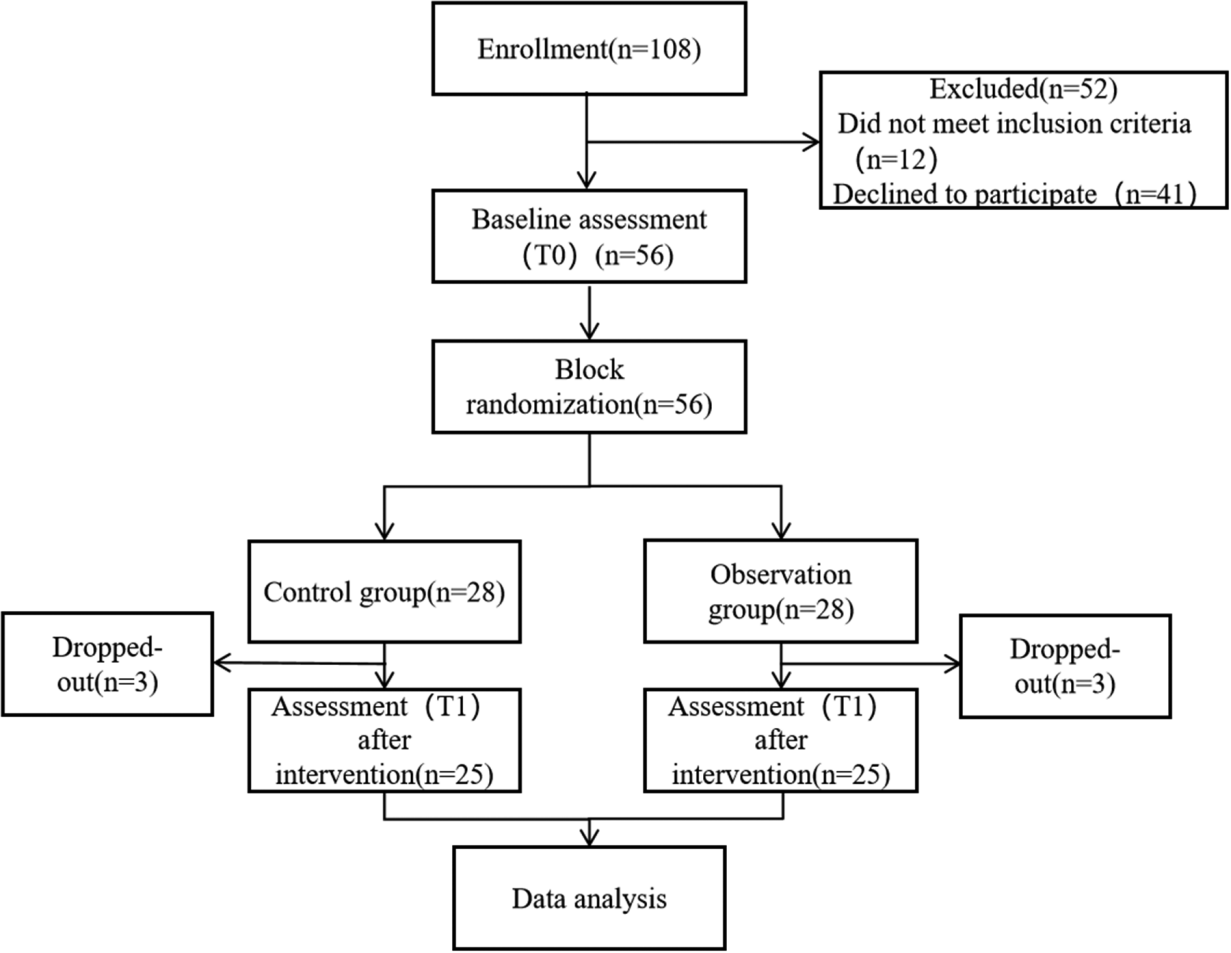
Flowchart of the study.

A total of 108 pediatric patients diagnosed with lobar pneumonia were assessed for eligibility. Among them, 52 were excluded, including 12 who did not meet the inclusion criteria and 41 who declined to participate. The remaining 56 participants underwent baseline assessment (T0) and were subsequently randomized into either the control group (*n* = 28) or the observation group (*n* = 28) using a permuted block randomization method. During the intervention period, three participants dropped out from each group due to various reasons, resulting in 25 participants in each group completing the post-intervention assessment (T1). All 50 participants who completed the study were included in the final data analysis. As shown in Figure 1.

### 3.2 Comparison of baseline data

There were no significant differences between the control and observation groups in terms of gender, age, disease duration, ethnicity, or infection site (*P* > 0.05). Laboratory parameters (CRP, WBC, PCT, LDH) and lung function indicators (FEV_1_, FVC, FEV_1_/FVC) also showed no statistically significant differences between the two groups (*P* > 0.05). As shown in Table 1.

**Table 1:** Baseline data comparison.

Variables		Control group (*n* = 25)	Observation group (*n* = 25)	*x^2^/Z*	*P*
Gender	Male	12 (48.00%)	10 (40.00%)	0.325	0.569
	Female	13 (52.00%)	15 (60.00%)		
Age (years)		6 (5,7)	6 (5.5, 7.5)	−1.149	0.250
Disease duration (days)		4 (3,5)	5 (3.5, 5)	−1.663	0.096
Infection site of lung lobe	Left	12 (48.00%)	11 (44.00%)	0.543	0.762
	Right	11 (44.00%)	13 (52.00%)		
	Bilateral	2 (8.00%)	1 (4.00%)		
CRP (mg/L)		24.13 ± 3.68	22.60 ± 4.35	1.345	0.185
WBC (×10^9^)		12.78 ± 3.85	13.45 ± 3.82	−0.619	0.539
PCT (ng/ml)		0.91 ± 0.13	0.88 ± 0.16	0.790	0.433
LDH (U/L)		266.68 ± 23.65	260.79 ± 23.00	0.891	0.377
FEV_1_ (L)		1.49 ± 0.20	1.38 ± 0.21	1.987	0.053
FVC (L)		1.89 ± 0.28	1.78 ± 0.27	1.405	0.167
FEV_1_/FVC (%)		78.99 ± 3.27	77.41 ± 3.23	1.722	0.092

CRP, C-reactive protein; WBC, white blood cell count; PCT, procalcitonin; LDH, lactate dehydrogenase; FEV_1_, forced expiratory volume in 1 s; FVC, forced vital capacity.

### 3.3 Comparison of duration of clinical symptoms between two groups

Compared with the control group, the observation group showed significantly shorter durations of fever (2.56 ± 1.12 vs. 3.84 ± 1.11 days, *P* < 0.001), cough (7.20 ± 1.04 vs. 10.16 ± 1.82 days, *P* < 0.001), lung rales (6.88 ± 1.45 vs. 9.80 ± 1.41 days, *P* < 0.001), antibiotic therapy (8.00 ± 1.66 vs. 11.48 ± 2.00 days, *P* < 0.001), and hospital stays (9.68 ± 2.38 vs. 12.16 ± 2.78 days, *P* = 0.001). These differences were statistically significant. As shown in Table 2.

**Table 2:** Comparison of duration of clinical symptoms between two groups.

Group	Duration of fever (d)	Duration of cough (d)	Duration of pulmonary crackles (d)	Course of antibiotic use (d)	Hospital stays (d)
Control group (*n* = 25)	3.84 ± 1.11	10.16 ± 1.82	9.80 ± 1.41	11.48 ± 2.00	12.16 ± 2.78
Observation group (*n* = 25)	2.56 ± 1.12	7.20 ± 1.04	6.88 ± 1.45	8.00 ± 1.66	9.68 ± 2.38
*t*	4.064	7.064	7.202	6.692	3.392
*P*	<0.001	<0.001	<0.001	<0.001	0.001
*Cohen's d*	−1.148	−1.997	−2.042	−1.893	−0.958

### 3.4 Comparison of pulmonary function indexes between two groups

Before intervention, no significant differences were observed between the two groups in FEV_1_, FVC, and FEV_1_/FVC (*P* > 0.05). After intervention, the observation group demonstrated significantly greater improvements in FEV_1_ (2.60 ± 0.14 vs. 2.44 ± 0.17 L, *P* = 0.001), FVC (2.87 ± 0.14 vs. 2.75 ± 0.20 L, *P* = 0.019), and FEV_1_/FVC ratio (90.51 ± 3.07% vs. 88.72 ± 2.77%, *P* = 0.035) compared to the control group. As shown in Table 3.

**Table 3:** Comparison of pulmonary function indexes between two groups.

Group	FEV_1_ (L)		FVC (L)		FEV_1_/FVC (%)	
	Before intervention	After intervention	Before intervention	After intervention	Before intervention	After intervention
Control group (*n* = 25)	1.49 ± 0.20	2.44 ± 0.17*	1.89 ± 0.28	2.75 ± 0.20*	78.99 ± 3.27	88.72 ± 2.77*
Observation group (*n* = 25)	1.38 ± 0.21	2.60 ± 0.14*	1.78 ± 0.27	2.87 ± 0.14*	77.41 ± 3.23	90.51 ± 3.07*
*t*	1.987	−3.691	1.405	−2.435	1.722	−2.167
*P*	0.053	0.001	0.167	0.019	0.092	0.035
Cohen's *d*		1.027		0.695		0.612

FEV_1_, forced expiratory volume in 1 s; FVC, forced vital capacity.

* Represents comparison with pre-intervention, *P* < 0.001.

### 3.5 Comparison of inflammatory markers between the two groups

Prior to intervention, there were no significant differences in CRP, WBC, PCT, and LDH levels between the control and observation groups (*P* > 0.05). After intervention, the observation group exhibited significantly greater reductions in inflammatory markers, including CRP (2.39 ± 0.38 vs. 7.40 ± 1.88 mg/L, *P* < 0.001), WBC (6.20 ± 1.21 vs. 8.05 ± 2.37 × 10^9^/L, *P* < 0.001), and PCT (0.13 ± 0.05 vs. 0.38 ± 0.12 ng/ml, *P* < 0.001), as well as in LDH (113.70 ± 14.90 vs. 164.77 ± 18.09 U/L, *P* < 0.001), compared to the control group. As shown in Table 4.

**Table 4:** Comparison of inflammatory markers between the two groups.

Group	CRP (mg/L)		WBC (×10^9^)		PCT (ng/ml)		LDH (U/L)	
	Before intervention	After intervention	Before intervention	After intervention	Before intervention	After intervention	Before intervention	After intervention
Control group (*n* = 25)	24.13 ± 3.68	7.40 ± 1.88*	12.78 ± 3.85	8.05 ± 2.37*	0.91 ± 0.13	0.38 ± 0.12*	266.68 ± 23.65	164.77 ± 18.09*
Observation group (*n* = 25)	22.60 ± 4.35	2.39 ± 0.38*	13.45 ± 3.82	6.20 ± 1.21*	0.88 ± 0.16	0.13 ± 0.05*	260.79 ± 2 3.00	113.70 ± 14.90*
*t*	1.345	13.087	−0.619	3.463	0.790	9.356	0.891	10.893
*P*	0.185	<0.001	0.539	<0.001	0.433	<0.001	0.377	<0.001
Cohen's *d*		−3.694		−0.983		−2.720		−3.082

CRP, C-reactive protein; WBC, white blood cell count; PCT, procalcitonin; LDH, lactate dehydrogenase.

* Represents comparison with pre-intervention, *P* < 0.001.

### 3.6 Comparison of treatment efficiency, parental satisfaction, and complication rate between the two groups

The observation group achieved significantly better outcomes than the control group. The total effective rate was higher in the observation group (100% vs. 84.00%, *P* = 0.037), with more cases of complete recovery (76.00% vs. 64.00%) and no ineffective cases, while the control group had a 16% non-response rate. Parental satisfaction was also greater in the observation group (96.00% vs. 76.00%, *P* = 0.042), with no reports of dissatisfaction compared to 12.00% in the control group. Furthermore, the incidence of adverse events was lower in the observation group (8.00% vs. 20.00%, *P* = 0.034), with only one case of bronchospasm (4.00%) and no other complications, whereas the control group had higher rates of laryngeal edema, pneumothorax, bronchospasm, and airway mucosal injury. As shown in Table 5.

**Table 5:** Comparison of treatment efficiency, parental satisfaction, and complication rate between the two groups.

Treatment efficiency					
Group	Cured	Improved		Ineffective	Total effective rate
Control group (*n* = 25)	16 (64.00%)	5 (24%)		4 (16%)	21 (84.00%)
Observation group (*n* = 25)	19 (76.00%)	6 (24.00%)		0 (0.00%)	25 (100%)
*F*					–
*P*					0.037
*RR (95% CI)*					1.190 (1.975–1.397)
Parental satisfaction					
Group	Very satisfied	Satisfied	Ordinary	Not satisfied	Overall satisfaction rate
Control group (*n* = 25)	15 (60.00%)	4 (16.00%)	3 (12.00%)	3 (12.00%)	19 (76.00%)
Observation group (*n* = 25)	23 (92.00%)	1 (4.00%)	1 (4.00%)	0 (0.00%)	24 (96.00%)
*x^2^*					–
*P*					0.042
*RR (95% CI)*					1.263 (0.999–1.597)
Complication rate					
Group	Laryngeal edema	Pneumothorax	Bronchospasm	Respiratory mucosal injury	Total complication rate
Observation group (*n* = 25)	0 (0.00%)	0 (0.00%)	1 (4.00%)	0 (0.00%)	2 (8.00%)
Control group (*n* = 25)	2 (8.00%)	1 (4.00%)	1 (4.00%)	4 (16.00%)	8 (20.00%)
*x^2^*					4.500
*P*					0.034
*RR (95% CI)*					0.250 (0.059–1.063)

RR, relative risk; CI, confidence interval.

## 4 Discussion

This randomized controlled trial evaluated the effectiveness of integrated medical and nursing management for pediatric lobar pneumonia. The findings suggest that, compared with conventional nursing care, the integrated intervention may offer benefits in reducing the duration of symptoms (fever, cough, pulmonary crackles), shortening antibiotic use and hospitalization, and improving pulmonary function and inflammatory markers. Higher rates of effective treatment and family satisfaction, along with a lower incidence of complications, were observed in the intervention group. However, these results should be interpreted with caution given the study's sample size and single-center design.

First, based on the results of this study, the integrated medical and nursing intervention was able to promote the relief of clinical symptoms in children within a short period, particularly in controlling high fever, severe cough, and lung inflammation absorption during the infection process. Notably, the effect sizes for these differences were large. This success can be attributed, to some extent, to the establishment of the daily joint rounds by the intervention team. Respiratory physicians and specialized nurses collaboratively developed individualized treatment plans and made timely adjustments to anti-infection strategies through dynamic assessments on the 1st, 3rd, and 7th days of hospitalization, which made disease control more targeted and timely. Additionally, lung function training programs, such as vibration positive expiratory pressure training and postural drainage, led by rehabilitation therapists, effectively improved alveolar ventilation, facilitated sputum expulsion, and accelerated the absorption of pulmonary lesions. The precise guidance on antibiotic usage paths provided by pharmacists also helped reduce the risk of antibiotic resistance. Through an interdisciplinary collaboration model, resources from specialized physicians, nurses, and rehabilitation therapists were integrated, reducing redundant work between disciplines. Nurses transitioned from passive executors to active participants, which enhanced their sense of responsibility and involvement. Furthermore, by participating in the management of complex cases, nurses gained professional growth opportunities and, compared to conventional nursing, experienced higher job pride.

Improvements in pulmonary function (FEV_1_, FVC, FEV_1_/FVC) were also more pronounced in the intervention group after treatment, with Cohen's d values indicating moderate to large effect sizes (0.612–1.027). This highlights the intervention's practical significance in promoting respiratory recovery beyond statistical significance. This finding aligns with the results of Khan et al. (21), who found that multidisciplinary collaboration improves lung ventilation function in the management of diffuse parenchymal lung diseases. Furthermore, the observation group demonstrated greater reductions in inflammation-related markers (CRP, WBC, PCT, LDH), which may indicate a more rapid control of inflammatory responses. This finding is broadly consistent with the report by Hart et al. (22), who suggested that family-centered care during the COVID-19 pandemic could facilitate faster recovery in patients with pneumonia. This perspective is also supported by Bellizzi et al. (23). Notably, the family nursing interventions in this study were somewhat innovative, such as the introduction of standardized video teaching, nebulization therapy simulation assessments, “parent classrooms”, and feedback mechanisms through the “Nursing Log”. These innovations not only improved the nursing capabilities of family members but also strengthened communication and trust between healthcare providers and families, further enhancing overall treatment adherence and satisfaction.

With respect to service quality, the satisfaction rate in the observation group was 96%, notably higher than the 76% observed in the control group. This suggests that integrated medical and nursing management may enhance the overall care experience, potentially by addressing both technical and emotional needs of families. The establishment of effective communication mechanisms between staff and families likely contributed to this result. Similar improvements in patient and family satisfaction with multidisciplinary collaborative care have been reported in other pediatric populations [Cowpe et al. (24)]. Furthermore, as demonstrated by Wei et al. (25), the integration of continuous multidisciplinary care and nutritional support can further improve patient outcomes. However, it is important to note that satisfaction is a subjective outcome and may be influenced by expectations, the novelty of the intervention, or other unmeasured factors.

Furthermore, the incidence of adverse events in the observation group was 8%, compared to 20% (RR = 0.250, 95% CI: 0.059–1.063) in the control group. Severe complications—including laryngeal edema, bronchospasm, pneumothorax, and respiratory mucosal injury—were observed only in the control group, while the observation group reported only one mild case of bronchospasm and no other serious events. This difference may be partly attributable to the use of a smart monitoring system in the intervention group, which provided real-time transmission of SpO_2_ and respiratory rate via bedside ECG monitors and incorporated a three-tiered alert threshold system to facilitate prompt clinical responses. Previous studies have shown that early warning systems can reduce delayed clinical responses and unexpected deterioration in pediatric patients [Bates et al. (26)], and the benefits of intelligent monitoring devices in critical care settings have been further supported by Malycha et al. (27) and Li et al. (28). However, the impact of such technology should be interpreted with caution, as other factors and the study's sample size may also have influenced the observed outcomes.

This study has several limitations. First, the observed effect sizes are substantial, the study's single-center design and limited sample size may have influenced the robustness of these findings. Second, although our study demonstrated significant short-term improvements in clinical symptoms, pulmonary function, and inflammatory markers, the long-term effects of the intervention remain unclear. In particular, data on pulmonary function at 3 or 6 months after discharge were not collected. Future studies should incorporate extended follow-up periods to better evaluate the durability of these clinical benefits. Third, the intervention was relatively complex and required considerable human and technical resources, which may restrict its feasibility in resource-limited settings. These aspects warrant further investigation and refinement in future studies. Furthermore, factors such as the quality of communication among team members, consistency in protocol implementation, and family engagement may have influenced the effectiveness of the intervention. These aspects warrant further investigation and refinement in future studies.

## 5 Conclusion

In conclusion, this study confirms the effectiveness and safety of integrated healthcare interventions in managing pediatric lobar pneumonia, demonstrating significant advantages over conventional care. The approach, which is child-centered, supported by multidisciplinary collaboration, and enhanced by intelligent tools, improves disease control, pulmonary function, reduces inflammation, increases satisfaction, and lowers complications.

## Data Availability

The original contributions presented in the study are included in the article/Supplementary Material, further inquiries can be directed to the corresponding author.
