# Supplementary material for: Integrated nursing and medical management improves outcomes in pediatric lobar pneumonia: a randomized controlled study
Source: Front Pediatr. 2025 Jul 28;13:1612618. doi: 10.3389/fped.2025.1612618 (PMC12336128; doi:10.3389/fped.2025.1612618)
Supplement: Supplementary file 1 [file Table1.docx]

Table 1 Multidisciplinary team collaboration for health management

| **Dimension** | **Implementation Content** | **Execution Standard** |
| --- | --- | --- |
| **Team Composition** | - One respiratory physician, two clinical nurses, one rehabilitation therapist, one clinical pharmacist, and one nutritionist | The team conducted joint rounds every two afternoon from 14:00 to 16:00 |
| **Individualized Treatment Plan** | - Stratification based on lung CT severity index (CTSI)  -Development of a precise anti-infection treatment plan based on pathogen detection | Ongoing assessment on the 1st, 3rd, and 7th day of hospitalization |
| **Respiratory Rehabilitation Training** | - Diaphragmatic breathing and pursed-lip breathing (twice daily, 10 minutes each)  - Postural drainage (in a 45° prone position, every 6 hours) | Use of a spirometer to monitor improvements in FEV1 |
| **Monitoring System** | - Continuous monitoring of SpO₂, respiratory rate, and heart rate/rhythm using bedside ECG monitors | Set alert thresholds:  -Level 1 Alert: SpO₂ <94% sustained for 3 minutes  -Level 2 Alert: SpO₂ <90% or respiratory rate >50 sustained for 2 minutes  -Level 3 Alert: Tachycardia (>180 bpm) accompanied by a >10% drop in SpO₂ from baseline sustained for 5 minutes |

Table 2 Family intervention

| **Module** | **Key Implementation Points** | **Quality Control** |
| --- | --- | --- |
| **Parent Skill Training** | - Standardized video tutorial (3D demonstration of the correct back-patting technique)  - Assessment of nebulization treatment technique via simulation (with a pass rate of 100%) | Skills are assessed by requiring three consecutive compliant operations |
| **Family Nursing Kit** | - Fingerclip-type pulse oximeter  - Respiratory training manual (illustrated version)  - Emergency contact card | A device usage test is conducted on the first day after admission |
| **Personalized Supervision** | Every Wednesday, a "Parent Education Class" is held, where specialized nurses provide answers to questions and review the "Nursing Log" | If missing records or abnormal data are found, parent education is conducted within 24 hours |
